# Knowledge mobilization activities to support decision-making by youth, parents, and adults using a systematic and living map of evidence and recommendations on COVID-19: protocol for three randomized controlled trials and qualitative user-experience studies

**DOI:** 10.1186/s13063-023-07067-9

**Published:** 2023-01-14

**Authors:** Rana Charide, Lisa Stallwood, Matthew Munan, Shahab Sayfi, Lisa Hartling, Nancy J. Butcher, Martin Offringa, Sarah Elliott, Dawn P. Richards, Joseph L. Mathew, Elie A. Akl, Tamara Kredo, Lawrence Mbuagbaw, Ashley Motillal, Ami Baba, Matthew Prebeg, Jacqueline Relihan, Shannon D. Scott, Jozef Suvada, Maicon Falavigna, Miloslav Klugar, Tamara Lotfi, Adrienne Stevens, Kevin Pottie, Holger J. Schünemann

**Affiliations:** 1 Michael G. DeGroote Cochrane Canada and McMaster GRADE Centres, Department of Health Research Methods, Evidence and Impact, McMaster University, 1280 Main St. W, Hamilton, Ontario L8S 4K1 Canada; 2 Child Health Evaluative Sciences, The Hospital for Sick Children Research Institute, Toronto, Ontario Canada; 3 Alberta Research Centre for Health Evidence, Department of Pediatrics, Faculty of Medicine and Dentistry, University of Alberta, Edmonton, Alberta Canada; 4 Schulich School of Medicine & Dentistry, Western University, London, Ontario Canada; 5 Cochrane Child Health, Department of Pediatrics, University of Alberta, Edmonton, Alberta Canada; 6 Department of Psychiatry, University of Toronto, Toronto, Ontario Canada; 7 Institute of Health Policy, Management and Evaluation, University of Toronto, Toronto, Ontario Canada; 8 Division of Neonatology, The Hospital for Sick Children, Toronto, Ontario Canada; 9 Five02 Labs Inc, Toronto, Ontario Canada; 10 Canadian Arthritis Patient Alliance, Toronto, Ontario Canada; 11 Postgraduate Institute of Medical Education and Research, Chandigarh, India; 12 Department of Internal Medicine, American University of Beirut, Beirut, Lebanon; 13 Cochrane South Africa, South African Medical Research Council, Cape Town, South Africa; 14 Division of Clinical Pharmacology, Department of Medicine and Division of Epidemiology and Biostatistics, Department of Global Health, Faculty of Medicine and Health Sciences, Stellenbosch University, Stellenbosch, South Africa; 15 Department of Anesthesia, McMaster University, Hamilton, Ontario Canada; 16 Department of Pediatrics, McMaster University, Hamilton, Ontario Canada; 17 Biostatistics Unit, Father Sean O’Sullivan Research Centre, St Joseph’s Healthcare, Hamilton, Ontario Canada; 18 Centre for Development of Best Practices in Health (CDBPH), Yaoundé Central Hospital, Yaoundé, Cameroon; 19 Division of Epidemiology and Biostatistics, Department of Global Health, Stellenbosch University, Cape Town, South Africa; 20 Centre for Addiction and Mental Health, Toronto, Ontario Canada; 21 Faculty of Nursing, University of Alberta, Edmonton, Alberta Canada; 22 Departments of Science and International Studies, St. Elizabeth University of Public Health and Social Science, Bratislava, Slovak Republic; 23 National Institute for Health Technology Assessment, Federal University of Rio Grande do Sul, Porto Alegre, Brazil; 24 Czech National Centre for Evidence-Based Healthcare and Knowledge Translation (Cochrane Czech Republic, Czech EBHC: JBI Centre of Excellence, Masaryk University GRADE Centre), Institute of Biostatistics and Analyses, Faculty of Medicine, Masaryk University, 625 00 Brno, Czech Republic; 25 Centre for Immunization Readiness, Public Health Agency of Canada, Ottawa, Canada; 26 Department of Family Medicine, Western University, London, Ontario Canada; 27 Department of Biomedical Sciences, Humanitas University, Milan, Italy

**Keywords:** eCOVID RecMap, COVID-19, Plain language recommendation, Standard language versions, Randomized controlled trial, Knowledge mobilization, Public engagement

## Abstract

**Introduction:**

The COVID-19 pandemic underlined that guidelines and recommendations must be made more accessible and more understandable to the general public to improve health outcomes. The objective of this study is to evaluate, quantify, and compare the public’s understanding, usability, satisfaction, intention to implement, and preference for different ways of presenting COVID-19 health recommendations derived from the COVID-19 Living Map of Recommendations and Gateway to Contextualization (RecMap).

**Methods and analysis:**

This is a protocol for a multi-method study. Through an online survey, we will conduct pragmatic allocation-concealed, blinded superiority randomized controlled trials (RCTs) in three populations to test alternative formats of presenting health recommendations: adults, parents, and youth, with at least 240 participants in each population. Prior to initiating the RCT, our interventions will have been refined with relevant stakeholder input. The intervention arm will receive a plain language recommendation (PLR) format while the control arm will receive the corresponding original recommendation format as originally published by the guideline organizations (standard language version). Our primary outcome is understanding, and our secondary outcomes are accessibility and usability, satisfaction, intended behavior, and preference for the recommendation formats. Each population’s results will be analyzed separately. However, we are planning a meta-analysis of the results across populations. At the end of each survey, participants will be invited to participate in an optional one-on-one, virtual semi-structured interview to explore their user experience. All interviews will be transcribed and analyzed using the principles of thematic analysis and a hybrid inductive and deductive approach.

**Ethics and dissemination:**

Through Clinical Trials Ontario, the Hamilton Integrated Research Ethics Board has reviewed and approved this protocol (Project ID: 3856). The University of Alberta has approved the parent portion of the trial (Project ID:00114894). Findings from this study will be disseminated through open-access publications in peer-reviewed journals and using social media.

**Trial registration:**

Clinicaltrials.gov NCT05358990. Registered on May 3, 2022

**Supplementary Information:**

The online version contains supplementary material available at 10.1186/s13063-023-07067-9.

## Administrative information

**Table:** 

Title	Knowledge mobilization activities to support decision-making by youth, parents, and adults using a systematic and living map of evidence and recommendations on COVID-19: protocol for three randomized controlled trials and qualitative user-experience studies
Trial Registration	The trials were registered on Clinicaltrials.gov on May 3^rd^, 2022, ID: NCT05358990. Link to registration: https://clinicaltrials.gov/ct2/show/NCT05358990?term=NCT05358990&draw=2&rank=1
Protocol version	May 3, 2022, version 1
Funding	“Canadian Institutes of Health Research” (CIHR), grant number: GA3-177732
Author Details	1. Michael G. DeGroote Cochrane Canada and McMaster GRADE Centres, McMaster University, Hamilton, Ontario, Canada, Department of Health Research Methods, Evidence and Impact, McMaster University, Hamilton, Ontario, Canada 2. Child Health Evaluative Sciences, The Hospital for Sick Children Research Institute, Toronto, Ontario, Canada 3. Alberta Research Centre for Health Evidence, Department of Pediatrics, Faculty of Medicine and Dentistry, University of Alberta, Edmonton, Alberta, Canada 4. Schulich School of Medicine & Dentistry, Western University, London, Ontario, Canada 5. Cochrane Child Health, Department of Pediatrics, University of Alberta, Edmonton, Alberta, Canada 6. Department of Psychiatry, University of Toronto, Toronto, Ontario, Canada 7. Institute of Health Policy, Management and Evaluation, University of Toronto, Toronto, Ontario, Canada 8. Division of Neonatology, The Hospital for Sick Children, Toronto, Ontario, Canada 9. Five02 Labs Inc, Toronto, Ontario, Canada 10. Canadian Arthritis Patient Alliance, Toronto, Ontario, Canada 11. Postgraduate Institute of Medical Education and Research, Chandigarh, India 12. Department of Internal Medicine, American University of Beirut, Beirut, Lebanon 13. Cochrane South Africa, South African Medical Research Council, Cape Town, South Africa 14. Division of Clinical Pharmacology, Department of Medicine and Division of Epidemiology and Biostatistics, Department of Global Health, Faculty of Medicine and Health Sciences, Stellenbosch University 15. Department of Anesthesia, McMaster University, Hamilton, ON, Canada 16. Department of Pediatrics, McMaster University, Hamilton, ON, Canada 17. Biostatistics Unit, Father Sean O'Sullivan Research Centre, St Joseph's Healthcare, Hamilton, ON, Canada 18. Centre for Development of Best Practices in Health (CDBPH), Yaoundé Central Hospital, Yaoundé, Cameroon 19. Division of Epidemiology and Biostatistics, Department of Global Health, Stellenbosch University, Cape Town, South Africa 20. Centre for Addiction and Mental Health, Toronto, Ontario, Canada 21. Faculty of Nursing, University of Alberta, Edmonton, Alberta, Canada 22. Departments of Science and International Studies, St. Elizabeth University of Public Health and Social Science, Bratislava, Slovak Republic 23. National Institute for Health Technology Assessment, Federal University of Rio Grande do Sul, Porto Alegre, Brazil 24. Czech National Centre for Evidence-Based Healthcare and Knowledge Translation (Cochrane Czech Republic, Czech EBHC: JBI Centre of Excellence, Masaryk University GRADE Centre), Institute of Biostatistics and Analyses, Faculty of Medicine, Masaryk University, 625 00 Brno, Czech Republic 25. Centre for Immunization Readiness, Public Health Agency of Canada, Ottawa, Canada 26. Department of Family Medicine, Western University, London, Ontario, Canada 27. Department of Biomedical Sciences, Humanitas University, Milan, Italy
Name and contact information for the trial sponsor	McMaster University is the sponsor of the trials and can be contacted through the principal investigator, Dr. Holger Schünemann at schuneh@mcmaster.ca.
Role of sponsor	The sponsor was responsible for the coordination and oversight of the trials.

## Strengths and limitations of this study

- We are following a multi-method approach: randomized controlled trials and qualitative interviews. The qualitative results will supplement and help explain our quantitative findings.
- This protocol is reported in accordance with the Standard Protocol Items: Recommendations for Interventional Trials (SPIRIT), which enhances transparency and completeness. The trials use previously validated outcomes from similar trials. This will strengthen the credibility of our results.
- Our study is testing an optimized plain language recommendation format, which makes our intervention relevant to our stakeholder groups, and is recruiting internationally, which ensures the inclusion of a diverse population. Recruitment will take place online using social media, and data will be collected using an online survey. This allows for self-selection and limits accessibility to those who have no or limited digital access, which in turn limits generalizability.
- While the recommendations are offered in multiple languages through the RecMap, the study is only testing English plain language recommendation summaries.

## Introduction

As health practitioners, policymakers, and the public are inundated with information, misinformation, and health recommendations about COVID-19, we have built a unique repository of trustworthy COVID-19 recommendations with funding from the Canadian Institutes of Health Research (CIHR) (covid19.recmap.org). The overarching goal of this COVID-19 RecMap effort is to identify all COVID-19 guidelines, assess their credibility and trustworthiness, and make their recommendations available and understandable to various stakeholder groups [1].

To enhance the understanding of health recommendations by the public, we developed a multi-stakeholder process to draft, edit, and publish plain language recommendations (PLRs) [2]. Until now, plain language versions of recommendations have been poorly explored, and our work on the RecMap indicates an absence of this critical knowledge mobilization tool for the general public, including youth, adults, and parents [3]. Existing trials on tailored recommendations presentations have been small and predated recent guidance on how to present guideline recommendations, targeted health professionals, or did not target the general public [4–7]. For example, a small trial with 84 mental health service users suggested an improved intention to follow recommendations when written in plain language [6]. Trial data also exists on specific aspects related to health information, like comparing alternative ways of presenting numerical or statistical information and formats of information sharing [4, 8–10]. However, trustworthy and comprehensive PLRs may need to include the clinical or public health background related to the topic, conflicts of interest, available research evidence, judgments that are made, the rationale for a recommendation, the actual recommendation, and implementation considerations. As addressed in Evidence to Decision (EtD) frameworks, these facets are deemed essential to inform decision-making and are used widely by various organizations [11–14].

A trial is necessary to empirically show that PLRs convey the intended messages from guidelines to broader populations and not only to the selected user groups that usually participate in ours and others’ research. Understanding and interpreting recommendations correctly are the essential prerequisites for the general public to become effective self-managers of their health and to ultimately optimize behaviors and related health outcomes [15]. Creating health information for the public that is accessible, reliable, and understandable is critical to scale science and evidence-based guidelines, which is especially important during the COVID-19 pandemic. We plan to undertake this trial, given the paucity of empirical evidence to guide our team and guideline developers as to how to best present PLRs and EtD facets to the public, which are critical to support their informed decision-making. This protocol describes the trials and details of the three leading trial sites.

## Objectives

The objective of this study is to compare end-users’ (youth, parents, and adult populations) understanding, accessibility and usability, satisfaction, intention to implement, and preference for COVID-19 recommendations when presented as plain language recommendation summaries (PLRs) versus standard language versions (SLVs). We hypothesize that there will be a difference in understanding of information between the two formats. We also aim to understand the reasons for their choices through our linked qualitative research.

## Methods

We will follow a multi-methods approach: randomized controlled trials (RCTs) with qualitative interviews among a subset of participants. The trials are pragmatic allocation-concealed, blinded superiority RCTs with at least 240 participants in each population (parents, adult, and youth). The trials were preceded by preparatory engagement work with Canadian youth advisors, a Canadian parent advisory group, and an international adult Cochrane Consumer group, herein referred to as stakeholder groups. The youth site’s preparatory engagement work was co-developed and implemented by two youth partners (MP and JR) in collaboration with LS, to ensure meaningful engagement efforts were embedded into the research process. We gathered input from stakeholders on COVID-19 priority topics and PLR content and format. Input from stakeholders helped us refine the PLR format to be evaluated with each population in the trial.

We will conduct qualitative interviews with a subset of the participants who completed the trials to help contextualize the digital user experience and understand the results of the trial.

Any amendments to this protocol will be made available via medRxiv.

### Randomized controlled trials

This protocol has been prepared in accordance with the Standard Protocol Items: Recommendations for Interventional Trials (SPIRIT) reporting guideline (Supplementary Material 1) [16].

### Design and setting

**Fig. 1:**
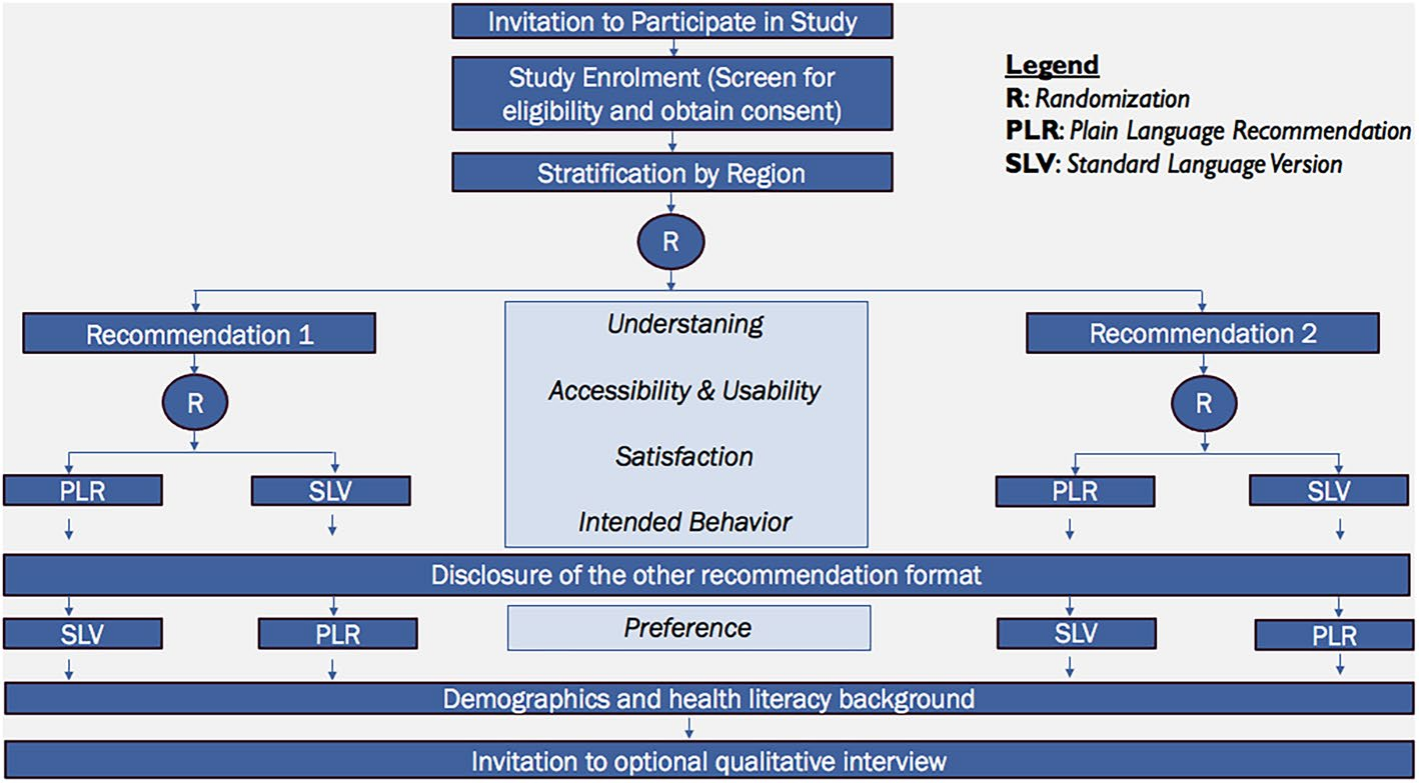
RCT flow diagram

There are three online survey links, one for each respective population. Each link contains eligibility questions, the consent form, and study information for youth, parents, or adults. Participants will access the online survey corresponding to their respective population to enter the trials and provide implied consent by participating in the survey. Using the concealed allocation code of the intervention platform, SurveyMonkey (surveymonkey.com), participants will select their geographic region. Participants from each geographic region will be randomized to one of two recommendation topics, and then they will be randomized to a PLR format (intervention) or a SLV format (control) of the recommendation (Fig. 1). The SLV is the original PDF guideline developed and published by an organization, such as the World Health Organization, from where the PLR information was extracted. Participants will complete an online survey consisting of Likert-scale questions, multiple-choice questions, open-ended questions, and demographic information. The survey is anonymous.

Following completion of the online survey, we will invite participants to take part in an optional qualitative interview that will allow them to reflect on their preference for the PLR or SLV format and help us understand the results of the quantitative survey. Confidentiality will be maintained by storing names and emails on a password-protected device and secure network to which only authorized study personnel have access to. If participants decide to provide their name and email address to participate in the interview, receive the study results, or enter the draw (for a chance to win one out of five $50 CAD gift cards for the adult and parents’ sites, one out of ten $25 CAD gift cards for the youth site), this personal information will not be linked to their survey answers.

This is not a clinical trial; thus, there are no foreseeable harms or health risks from participating. However, there is a possibility that participants may experience distress once they better understand the uncertainty in the underlying evidence, even if the recommendation itself is judged trustworthy. We provide the email address and contact information of the principal investigator and research coordinator. Participants can contact us to report any discomfort or seek further information.

### Participants

Each of the three leading sites will recruit participants from one of the three population groups: adults, parents, or youth. SickKids Research Institute will lead the recruitment of youth, Western University will lead the recruitment of adults, and The University of Alberta will lead the recruitment of parents. Global recruitment efforts will take place via social media. Coordination and oversight of the trials rest with the research team at McMaster University (RC and HJS). Our study is also open for other sites who are interested in participating as active recruitment sites for one or more populations. For example, St. Elizabeth University in Slovakia is joining as an active recruitment site for all three populations, with a commitment to recruit up to 60 participants.

#### Selection criteria

In this study, adults are defined as individuals self-reported to be 21 years of age or older, youth are defined as individuals self-reported to be between the ages of 15 and 24 years [17], and parents are defined as individuals self-reported to be 18 years or above and are a parent or legal guardian of a child under 18 years old. Participants can be from any country. For all three populations, participants will need to be able to read and understand English. We expect variation in the English language proficiency and health literacy level among participants. Therefore, when collecting demographic information, questions related to language proficiency and familiarity with COVID-19 health information will be included to understand the participant’s level of health literacy. We may adjust for these variables in our analysis if needed, although we expect them to be reasonably balanced between the SLV and PLR groups due to randomization.

#### Recruitment

Each leading site will run recruitment for the different population groups separately yet with similar and overlapping strategies. We will recruit English-speaking (mother tongue or fluent) participants globally through our experienced RecMap investigator team (https://covid19.recmap.org/about), the Cochrane Consumer Network and various Cochrane networks, guideline co-authors who interact with youth (including the international Young Persons’ Advisory Groups), parents, and adult citizens. We plan to run public recruitment campaigns through identified youth, adult, and parent organizations’ social media platforms (e.g., organizations’ accounts on Twitter, Instagram, and Facebook) and newsletters. Each leading site will be responsible for monitoring its own recruitment, though all investigators will support overall recruitment for the trials (e.g., if recruitment falls behind for one of the populations, other sites will help support recruitment for that group). Surveys will be administered through SurveyMonkey, where participants are screened for eligibility prior to allocation. Previous studies suggest that the target recruitment rates for such trials are achievable in less than 3 months, and we will recruit at least 240 participants per group [18–20].

The average survey completion time is estimated to be 15–25 min, based on stakeholder feedback and pilot testing. As suggested by our stakeholders in the preparation of this trial, we will be inviting participants who complete the survey to enter a draw for a chance to win one of five $50 CAD gift cards for the adult and parents’ populations. The youth group will invite youth participants to enter a draw for one of ten gift cards valued at $25 CAD. This is offered as a gesture and appreciation for completing the survey.

### Intervention and comparison

**Fig. 2:**
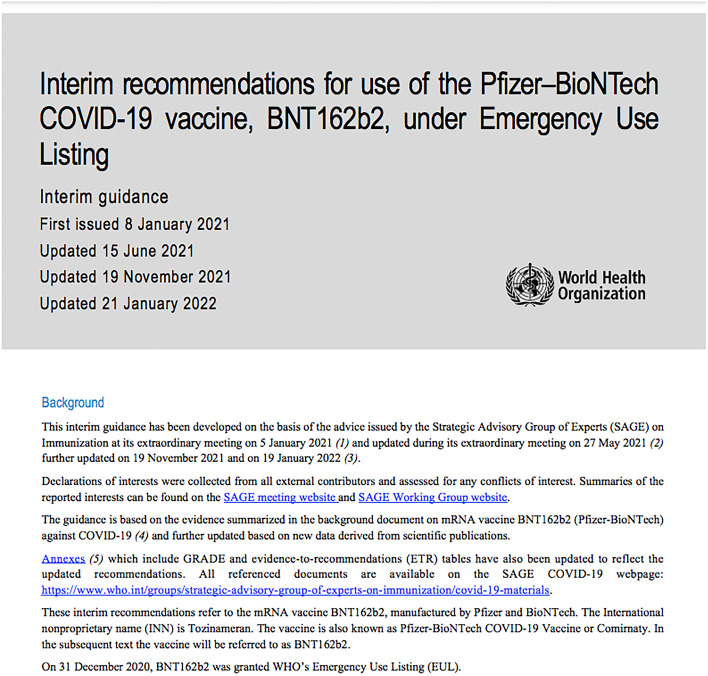
SLV format for youth and adult participants

**Fig. 3:**
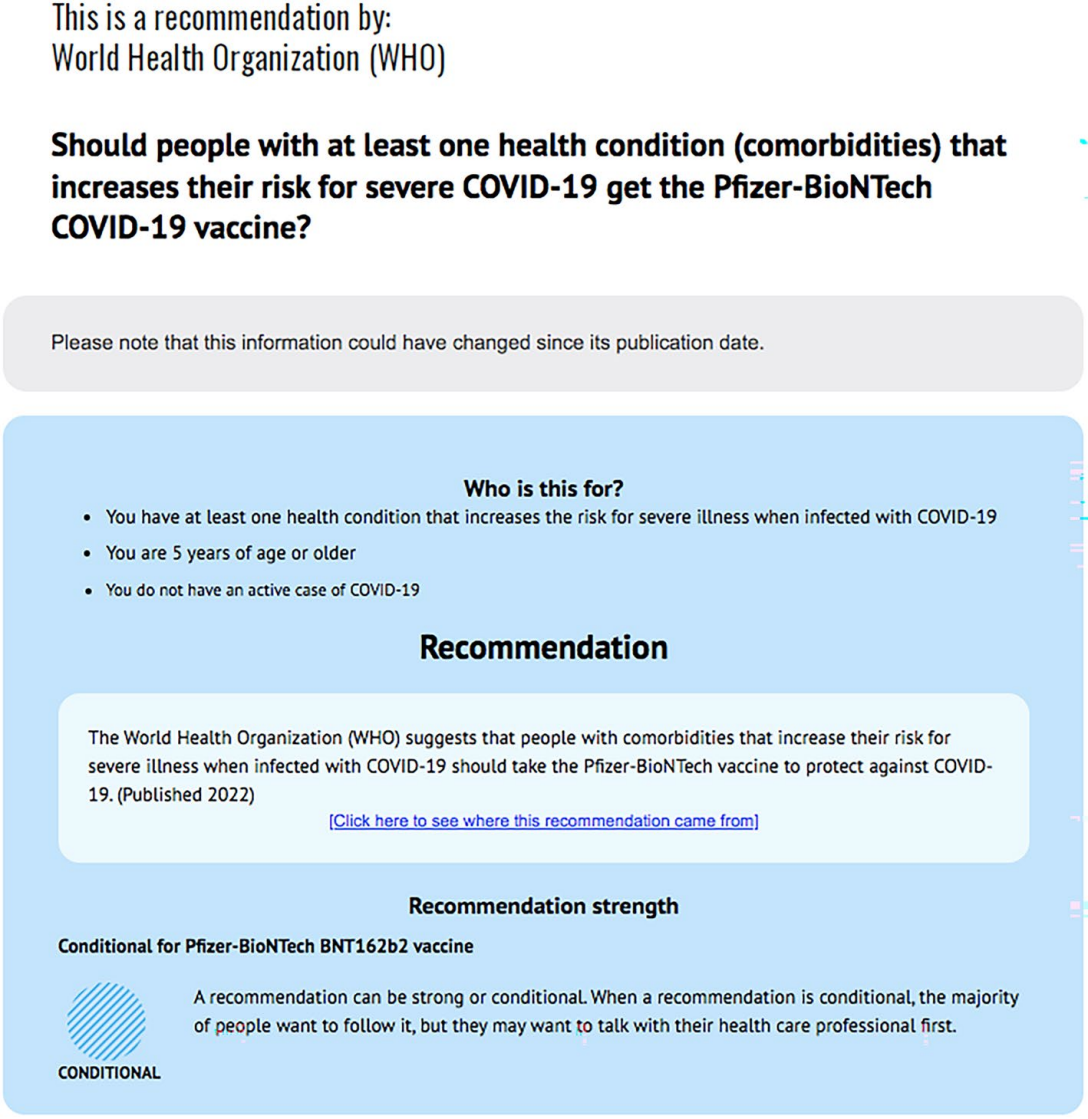
Example of a plain language recommendation summary format for adult participants

In these trials, the intervention is the PLR, and the comparison is the SLV. Figure 2 shows an example of the SLV format of a recommendation that will be used with the adult and youth participants, and Fig. 3 shows an example of a PLR format that will be presented to adult participants. Since this is a non-clinical trial, receiving either the intervention or the control will not require alteration to any part of the participant’s life. Additionally, we do not have special criteria for discontinuing or modifying the allocated intervention.

Through our pre-trial engagement work, we gathered input from youth (via online workshops) and from adult stakeholders (via online meetings) on high-priority COVID-19 topics and on PLR content and format. Input from youth and adult stakeholders allowed us to choose two recommendations for the trials and refine the PLR format. For the parents’ population, the PLRs were refined from collected feedback during online meetings with parent groups.

All PLRs for the trials were developed from the same template and refined using stakeholder feedback. The youth and parent PLRs’ design and formatting are the same, as these groups gave similar feedback, while the adult PLRs are slightly different.

In the trials, we will ask participants to read one format of a recommendation (PLR or SLV) to which they have been randomly allocated and then answer the online survey that takes an average of 15–25 min to complete.

### Outcomes

#### Primary outcome

##### Understanding

We define understanding as the comprehension of key guideline content (e.g., year of publication, intent of recommendation, direction of recommendation, etc.). This outcome will be measured using seven multiple-choice questions about key concepts in the recommendation with four to six response options for each question and only one correct answer (total minimum score of 0, maximum score of 7). Based on previous work, we aim to detect a difference of 10% in understanding between groups which we consider an important difference (an average difference of 1 correct answer) [18, 19, 21–23].

#### Secondary outcomes

##### Accessibility and usability

We define accessibility and usability as the ability to find/access and use the presented information. This outcome considers the three following domains: (1) how easy it was to find information, (2) how easy it was to understand the information (perception), and (3) whether the information was presented in a way that could be helpful for making an informed health decision. Participants will be asked to indicate the degree of agreement with six statements, measured using the 7-point Likert scale (1 being strongly disagree, 4 being neutral, and 7 being strongly agree). We will also include 1 open-ended question to get additional input on accessibility and usability. The outcome “overall accessibility/usability of information” will be measured using the average of the 7-point Likert-type scale questions.

##### Satisfaction 

We define satisfaction as participants’ impression of the recommendation’s presentation. We will ask participants about their level of satisfaction with different features of the format (e.g., order of information, length of document, etc.) [24]. Participants will be asked to indicate the degree of satisfaction with three questions measured using the original 7-point Likert-type scale (1 being very dissatisfied, 4 being neutral, and 7 being very satisfied). We will also include two open-ended questions to get their input on what they liked and disliked in the format.

##### Intended behavior

We define intended behavior as participants’ intention in adopting and following the shared recommendation. We will ask participants if they have already followed the recommendation and have them respond with yes, no, unsure, prefer not to answer, or not applicable. Subsequently, we will ask how likely it is that they will follow the recommendation (if they have not already) and share the recommendation with others, through two questions measured using a 7-point Likert-type scale (1 being very unlikely, 4 being neutral, and 7 being very likely).

##### Preference

We define preference as a greater liking of one format over the other (PLR or SLV). After participants complete the tasks in the group they are randomized to, they will be asked to compare the PLR and the SLV formats. Participants will review the alternative format and indicate their preference for one of the two formats using a 7-point Likert-type scale (1 being strongly preferring the SLV, 4 being the same preference for both, and 7 being strongly preferring PLR). Participants will also have the chance to explain their choice in a text box.

### Stratification

We will stratify our participants by region: Africa, Americas, Eastern Mediterranean, Europe, South-East Asia, and Western Pacific. We have not set a target sample per strata. The rationale behind stratification is to ensure an equal chance for participants from the same region to be randomized to either the intervention or control (as opposed to having all participants from Europe, for example, being randomized to the PLR format).

### Randomization

After stratification, participants will be randomly assigned in a 1:1 ratio to recommendation topic 1 or 2, then will be randomized again in a 1:1 ratio to the PLR or the SLV arms (see Fig. 1). Randomization will ensure that recommendation formats will be equally distributed across participants to get balanced judgments on outcomes.

### Allocation concealment

The allocation sequence is concealed using SurveyMonkey® software based on a commercial, but unknown algorithm without a pre-identified sequence.

### Blinding

Participants will know that the trials are testing different formats to present health recommendations but will be blinded with respect to what formats are being compared and to the group to which they are allocated. Participants will not be aware of their random allocation to the PLR or SLV format until disclosure. Thus, participants will be blinded for all outcomes except the secondary outcome of preference. We will blind data analysts involved in data interpretation and manuscript writing to reduce bias in data analysis and interpretation [25, 26]. To reduce bias, we will draft each population’s manuscript with general group labels (i.e., group A vs B) and agree on the final interpretation before the group allocation (optimized PLR or SLV) is revealed.

### Sample size calculation

The sample size was calculated using the primary outcome of understanding. For this two-sided (*α* = 0.05) superiority analysis, these computations were made based on a *t*-test with the null hypothesis that there is no difference between the PLR version and the SLV in understanding of information.

- *H*_*0*_*:* PLR version = SLV
- *H*_*1*_*:* PLR version ≠ SLV

We will determine whether we can reject the null hypothesis of no difference in understanding between the formats of presenting recommendations. We assume a correct response rate (regarding the understanding of information) of 75% in the PLR arm and 60% in the control arm [relative risk =1.25] based on a recent RCT and prior trials on plain language summaries (PLS) of summary of findings table [19, 23]. With typical alpha (0.05) and beta (0.8) parameters and an allocation ratio of 1:1, 240 participants (120 in each arm) would be required (Stata/SE 16.0) [27]. Assuming 15% non-completion, we will recruit 282 participants from each population and adjust this number if non-completion is higher.

Since we are using two different recommendation topics with each population, we might observe an interaction between the recommendation topic and our outcome of understanding. Thus, when we reach half of our intended sample size, we will conduct an interim analysis for a possible interaction effect. If the data suggests that there might be an interaction, we will consider modifying our initial sample size. This new sample size cannot be determined in advance since we cannot anticipate the magnitude of the potential interaction effect, but it will be determined based on published guidance on power calculations for credible subgroup effects [28]. If needed, we will submit an amendment that explains the new sample size and need for additional recruitment based on the magnitude of the interaction effect identified. In summary, an enhanced sample size, if indicated and feasible, will allow us to conduct a more meaningful analysis by exploring the results by topic of recommendation.

### Consultation and pilot testing

We will use the same survey template for all populations; however, the “understanding” questions will be appropriately different for each recommendation. We pilot tested the surveys during the pre-trial engagement work to gather feedback from Canadian youth advisors, Cochrane Consumer Network, adult stakeholders, and parent stakeholders. We had 10 youth, 29 adults, and 5 parents pilot test the survey and provide feedback on the time to complete the survey, length of the survey, clarity of questions, Internet difficulties, and any other comments to enhance the survey experience. Revisions were made to the surveys using pilot test feedback until no errors or inconsistencies were detected and the surveys were easily understood. The survey language used for each population is tailored based on the feedback from stakeholders. Pilot test participant results will not be included in the final analysis.

### Statistical analysis

Only participants who submit a complete good quality survey will be included in the analysis. We will exclude all incomplete, speeding (less than 6 min to completion), and straight-lining responses. Each population’s results will be analyzed separately, and we will explore subgroup effects within each group. These subgroups are the topic of recommendation, health literacy level, and English proficiency level of participants. We will also pool the results across populations and conduct a meta-analysis for our primary outcome of “understanding” and other secondary outcomes. In the final analysis, after pooling the results, we will explore for potential interaction related to the recommendation topic and participants’ health literacy level. We will conduct the analyses using IBM SPSS® (Statistical Package for Social Sciences) version 23.

#### Descriptive analysis

We will summarize participant baseline characteristics and outcomes using means and standard deviations (SD) for continuous variables and proportions for categorical variables.

#### Inferential analysis

We will perform a primary analysis including all randomized participants. We will exclude participants who take less than 6 min to complete the survey. For the outcome of understanding, we will use *χ*^2^ tests and risk difference with 95% CIs to compare the proportion of correct responses between groups. For the outcomes of accessibility and satisfaction, we will use *t*-tests and mean differences 95% confidence intervals (95% CIs) to compare means and SDs between the intervention and control groups. Finally, we will present preference as mean (SD) overall and for both trial arms. Skewness, Shapiro-Wilk tests, and histograms will be used to evaluate whether the distribution was shifted toward the same preference in both groups. Levene’s test of equal variances will be used for all *t*-tests and degrees of freedom will be adjusted for *p* < 0.05. We will report all *p*-values to three decimal places, with values less than 0.001 reported as < 0.001. We will consider statistical significance at *p* < 0.05. Furthermore, we will conduct a meta-analysis across the three trials by combining results of the three trials. Our a priori hypothesis is that there is no difference across the three populations despite the differences in population characteristics and slight difference in the presentation formats for each population. However, we will explore differences between trials applying the GRADE approach to evaluating inconsistency which includes the chi-square test, the *I*^2^ value, the overlap in confidence intervals, and the differences in the point estimates of effect for all outcomes [29].

### Qualitative methods

We will interview a sample of survey participants to explore their user experience with two recommendation formats. These semi-structured interviews will help us contextualize the results of the survey and understand participants’ preference for receiving the information.

#### Selection of participants

At the end of each survey, participants will be invited to contribute to a virtual, one-on-one, semi-structured interview, conducted by the research coordinator for each leading site. Interested participants will voluntarily provide their email address at the end of the survey, and the research coordinator will follow up with them through email.

#### Interviews

We will invite individuals from the list of participants who have agreed to participate in the qualitative interviews. For the parents’ population, we will use purposive sampling (inviting participants based on region, ethnicity, gender, and topic of recommendation). For the adult and youth populations, we will not use a purposive sample; however, we will collect demographic data from the participants at the time of the interview. All interview participants will be compensated for their time with a gift card valued at $25 CAD.

Prior to the interview, participants will be provided with the consent form for the semi-structured interviews. Following that, a Zoom invite will be shared with the participant. Prior to the start of the interview, the research coordinator conducting the interview will obtain verbal consent from the participant for participating in the interview and for the interview to be audio-recorded. If the participant chooses to leave their video camera on during the interview, consent will be acquired for video-recording the interview. Consent will be documented on a log by the coordinator. If participants decline recording of the interviews, we will not move forward with the interview. We will transcribe interview recordings verbatim and de-identify all interview transcripts.

Interviews will be conducted in English by trained interviewers and will take approximately 30–60 min. Interviews will be guided by open-ended questions that cover the seven facets of the honeycomb model to verify user’s experiences with the different recommendation formats: usefulness, usability, findability, accessibility, desirability, credibility, and value [30]. We will pilot test the interview guide within the three populations to refine the questions and language based on the populations’ unique needs prior to the interviews.

#### Data management

All recordings will be stored on a password-protected device and secure network and accessed only by members of the research team. The recordings will be kept for 5 years, at which point they will be destroyed. All transcribed recordings will be reviewed and verified by the interviewer or a second research team member prior to being de-identified. NVivo software (QSR, 2018) will be used for data coding and management.

#### Data analysis

**Fig. 4:**
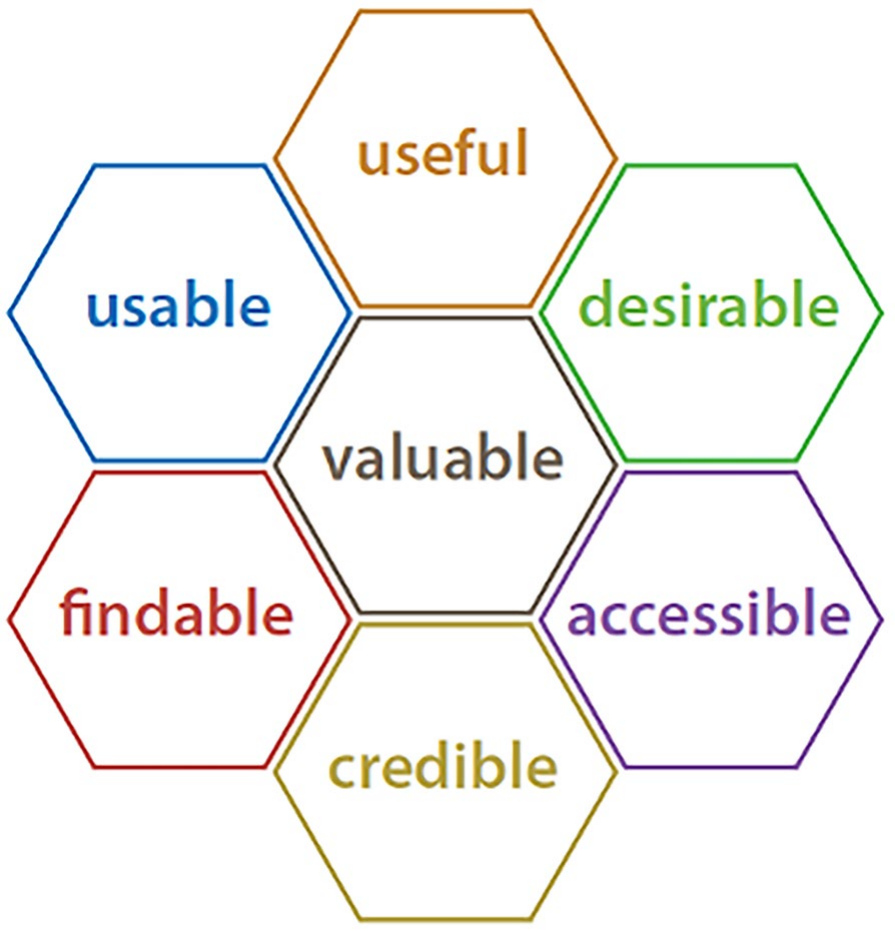
The honeycomb model of user experience

Qualitative data analysis will follow a hybrid inductive/deductive method [31]. This method allows for flexibility to utilize an established framework during analysis (deductive) as well as identify codes that emerge from the data (inductive). The 7 elements of the honeycomb model for user experience (Fig. 4) informed the creation of the interview guide and were adapted and used as the initial model for analysis (see Supplementary Material 2 for the interview guide) [30]. The seven elements of the model will guide coding with inductive coding of data that extends beyond the model.

Data collection and analysis will occur iteratively [32]. The analysis will follow six steps: (1) data cleaning-comparing the transcript with the recording and doing the cleaning to ensure alignment, (2) generating initial codes using line-by-line coding, (3) searching for themes guided by the honeycomb model, (4) reviewing the themes and generating a “thematic map,” (5) defining and naming themes guided by the honeycomb model, and (6) writing the report. Data collection will occur until saturation is reached [33].

We will develop joint displays, explicitly merging the results from the three populations’ data sets through a side-by-side comparison to assess how findings are aligned among the three data sets [34].

#### Rigor

Strategies for maintaining rigor [35] will be used such as utilize a detailed study log and audit trail to ensure transparency. A coding system will be utilized and refined throughout the iterative data collection and analysis stages and each site will have a primary coder and a secondary coder to code approximately 10% of the transcripts and compare to maintain intra-rater reliability [35]. We will use iterative member checking, reflecting back statements made during interviews to participants for clarity and understanding.

We will also use our trial survey data to triangulate our findings with survey responses. Our analysis team will reflect on and disclose our own digital experiences and consider this in our iterative analysis process.

## Reporting

We will report our quantitative findings according to the Consolidated Standards of Reporting Trials (CONSORT) reporting guidelines and qualitative findings following the Consolidated criteria for reporting qualitative research (COREQ) [36, 37]. For the quantitative results, we will pool the results across the three populations and conduct a meta-analysis for our primary outcome of “understanding” and other secondary outcomes. We will explore results for each population separately as well as compare results across populations.

Finally, we will crossmatch and compare the results of the qualitative interviews with those of the quantitative surveys and try to better understand and contextualize the RCT findings. Based on the results, we will discuss possible changes to the PLR format.

## Discussion

Guideline developers typically create recommendations for health professionals or health agencies [1]. However, particularly in the context of COVID-19 and any future pandemics or topics of high public interest, the general public must have access to health recommendations that they can understand and trust [38]. For example, public understanding and acceptance is required for the implementation and uptake of many public health recommendations such as vaccination, using facial masks, and social distancing. Furthermore, parents are making decisions for children who cannot make decisions for themselves, and they need trustworthy recommendations specific to this unique population (e.g., vaccine safety and efficacy for children). On the other hand, youth access health recommendations about COVID-19 and make independent decisions about implementing public health measures, like social distancing and face mask use [3].

It has become crucial to engage end-users in shaping behaviors and activities that will eventually affect them and their communities. Their involvement will improve the relevance, usefulness, and transparency of guidelines [39]. More importantly, it will ensure that these guideline products are understandable and acceptable. We believe that when the public is presented with plain language recommendations, they will have a better understanding of guideline recommendations. These trials are designed with the input of end-users from three populations: youth, adults, and parents. Results will inform the best ways to make recommendations understandable and accessible to the public, thus increasing the public’s confidence in science and evidence uptake.

## Trial status

Protocol version 1, May 3, 2022

Started recruitment: May 26, 2022

Estimated recruitment completion: October 31, 2022

## Supplementary Information

**Additional file 1: Supplementary Material 1.** SPIRIT Checklist.

**Additional file 2: Supplementary Material 2.** Semi-structured interview guide.

**Additional file 3: Supplementary Material 3.** Survey Consent Form Sample.

**Additional file 4: Supplementary Material 4.** Interview Consent Form Sample.

## Data Availability

Anonymous survey data will be publicly available from an open-access online repository to be chosen within 2 years of data collection. The datasets analyzed during the current study and statistical code are available from the corresponding author on reasonable request, as is the full protocol.

## Abbreviations

CAD: Canadian Dollars
CIHR: Canadian Institutes of Health Research
CONSORT: Consolidated Standards of Reporting Trials
COREQ: Consolidated Criteria for Reporting Qualitative Research
COVID-19: Coronavirus disease of 2019
GIN: Guidelines International Network
HiREB: Hamilton Integrated Research Ethics Board
KM: Knowledge mobilization
PLR: Plain language recommendation
RecMap: COVID-19 Living Map of Recommendations and Gateway to Contextualization
RCT: Randomized controlled trial
SLV: Standard language version

## References

[1] Lotfi T, Stevens A, Akl EA, Falavigna M, Kredo T, Mathew JL (2021). Getting trustworthy guidelines into the hands of decision-makers and supporting their consideration of contextual factors for implementation globally: recommendation mapping of COVID-19 guidelines. J Clin Epidemiol.

[2] Pottie K, Smith M, Matthews M, et al. A multistakeholder development process to prioritize and translate COVID-19 health recommendations for patients, caregivers and the public. A case study of the COVID-19 Recommendation Map. J Clin Epidemiol. 2021; (in review). 10.1016/j.jclinepi.2022.04.012 PMC9055415 35500815

[3] Dietl B. Google analytics data of the covid19.recmap.org Retrieved 07 April, 2021. 2021.

[4] Akl EA, Oxman AD, Herrin J, Vist GE, Terrenato I, Sperati F (2011). Using alternative statistical formats for presenting risks and risk reductions. Cochrane Database Syst Rev.

[5] Ince P, Tai S, Haddock G (2015). Using plain English and behaviourally specific language to increase the implementation of clinical guidelines for psychological treatments in schizophrenia. J Ment Health.

[6] Michie S, Lester K (2005). Words matter: increasing the implementation of clinical guidelines. BMJ Qual Saf.

[7] Shekelle PG, Kravitz RL, Beart J, Marger M, Wang M, Lee M (2000). Are nonspecific practice guidelines potentially harmful? A randomized comparison of the effect of nonspecific versus specific guidelines on physician decision making. Health Serv Res.

[8] Anzinger H, Elliott SA, Hartling L (2020). Comparative usability analysis and parental preferences of three web-based knowledge translation tools: multimethod study. J Med Internet Res.

[9] Buljan I, Tokalić R, Roguljić M, Zakarija-Grković I, Vrdoljak D, Milić P (2020). Framing the numerical findings of Cochrane plain language summaries: two randomized controlled trials. BMC Med Res Methodol.

[10] Buljan I, Malički M, Wager E, Puljak L, Hren D, Kellie F (2018). No difference in knowledge obtained from infographic or plain language summary of a Cochrane systematic review: three randomized controlled trials. J Clin Epidemiol.

[11] Moberg J, Oxman AD, Rosenbaum S, Schünemann HJ, Guyatt G, Flottorp S (2018). The GRADE Evidence to Decision (EtD) framework for health system and public health decisions. Health Res Policy Syst.

[12] Parmelli E, Amato L, Oxman AD, Alonso-Coello P, Brunetti M, Moberg J (2017). GRADE Evidence to Decision (EtD) framework for coverage decisions. Int J Technol Assess Health Care.

[13] Alonso-Coello P, Schünemann HJ, Moberg J, Brignardello-Petersen R, Akl EA, Davoli M (2016). GRADE Evidence to Decision (EtD) frameworks: a systematic and transparent approach to making well informed healthcare choices. 1: Introduction. BMJ..

[14] Alonso-Coello P, Oxman AD, Moberg J, Brignardello-Petersen R, Akl EA, Davoli M (2016). GRADE Evidence to Decision (EtD) frameworks: a systematic and transparent approach to making well informed healthcare choices. 2: clinical practice guidelines. BMJ..

[15] Schulz PJ, Nakamoto K (2013). Health literacy and patient empowerment in health communication: the importance of separating conjoined twins. Patient Educ Couns.

[16] Chan A-W, Tetzlaff JM, Gøtzsche PC, Altman DG, Mann H, Berlin JA (2013). SPIRIT 2013 explanation and elaboration: guidance for protocols of clinical trials. BMJ..

[17] World Health O. Adolescent Health: World Health Organization; 2021.

[18] Carrasco-Labra A, Brignardello-Petersen R, Santesso N, Neumann I, Mustafa RA, Mbuagbaw L, et al. Improving GRADE evidence tables part 1: a randomized trial shows improved understanding of content in summary of findings tables with a new format. J Clin Epidemiol. 2016;74:7–18. 10.1016/j.jclinepi.2015.12.007 26791430

[19] Vandvik PO, Santesso N, Akl EA, You J, Mulla S, Spencer FA (2012). Formatting modifications in GRADE evidence profiles improved guideline panelists comprehension and accessibility to information. A randomized trial. J Clin Epidemiol.

[20] Santesso N, Rader T, Nilsen ES, Glenton C, Rosenbaum S, Ciapponi A (2015). A summary to communicate evidence from systematic reviews to the public improved understanding and accessibility of information: a randomized controlled trial. J Clin Epidemiol.

[21] Carrasco-Labra A, Brignardello-Petersen R, Santesso N, Neumann I, Mustafa RA, Mbuagbaw L (2015). Comparison between the standard and a new alternative format of the Summary-of-Findings tables in Cochrane review users: study protocol for a randomized controlled trial. Trials..

[22] Glenton C, Santesso N, Rosenbaum S, Nilsen ES, Rader T, Ciapponi A (2010). Presenting the results of Cochrane Systematic Reviews to a consumer audience: a qualitative study. Med Decis Mak.

[23] Rosenbaum SE, Glenton C, Oxman AD (2010). Summary-of-findings tables in Cochrane reviews improved understanding and rapid retrieval of key information. J Clin Epidemiol.

[24] Santesso N, Glenton C, Dahm P, Garner P, Akl EA, Alper B (2020). GRADE guidelines 26: informative statements to communicate the findings of systematic reviews of interventions. J Clin Epidemiol.

[25] Järvinen TL, Sihvonen R, Bhandari M, Sprague S, Malmivaara A, Paavola M (2014). Blinded interpretation of study results can feasibly and effectively diminish interpretation bias. J Clin Epidemiol.

[26] Schünemann HJ, Armstrong D, Degl'innocenti A, Wiklund I, Fallone CA, Tanser L (2004). A randomized multicenter trial to evaluate simple utility elicitation techniques in patients with gastroesophageal reflux disease. Med Care.

[27] Faul F, Erdfelder E, Lang A-G, Buchner A (2007). G* Power 3: a flexible statistical power analysis program for the social, behavioral, and biomedical sciences. Behav Res Methods.

[28] Burke JF, Sussman JB, Kent DM, Hayward RA (2015). Three simple rules to ensure reasonably credible subgroup analyses. BMJ..

[29] Guyatt GH, Oxman AD, Kunz R, Woodcock J, Brozek J, Helfand M (2011). GRADE guidelines: 7. Rating the quality of evidence—inconsistency. J Clin Epidemiol.

[30] Morville P (2004). User experience design.

[31] Fereday J, Muir-Cochrane E (2006). Demonstrating rigor using thematic analysis: a hybrid approach of inductive and deductive coding and theme development. Int J Qual Methods.

[32] Braun V, Clarke V (2006). Using thematic analysis in psychology. Qual Res Psychol.

[33] Saunders B, Sim J, Kingstone T, Baker S, Waterfield J, Bartlam B (2018). Saturation in qualitative research: exploring its conceptualization and operationalization. Qual Quant.

[34] Haynes-Brown TK, Fetters MD (2021). Using joint display as an analytic process: an illustration using bar graphs joint displays from a mixed methods study of how beliefs shape secondary school teachers’ use of technology. Int J Qual Methods.

[35] Morse JM (2015). Critical analysis of strategies for determining rigor in qualitative inquiry. Qual Health Res.

[36] Moher D, Hopewell S, Schulz KF, Montori V, Gøtzsche PC, Devereaux P (2012). CONSORT 2010 explanation and elaboration: updated guidelines for reporting parallel group randomised trials. Int J Surg.

[37] Tong A, Sainsbury P, Craig J (2007). Consolidated criteria for reporting qualitative research (COREQ): a 32-item checklist for interviews and focus groups. Int J Qual Health Care.

[38] Schünemann HJ, Santesso N, Vist GE, Cuello C, Lotfi T, Flottorp S (2020). Using GRADE in situations of emergencies and urgencies: certainty in evidence and recommendations matters during the COVID-19 pandemic, now more than ever and no matter what. J Clin Epidemiol.

[39] Petkovic J, Riddle A, Akl EA, Khabsa J, Lytvyn L, Atwere P (2020). Protocol for the development of guidance for stakeholder engagement in health and healthcare guideline development and implementation. Syst Rev.

